# Correlative super-resolution analysis of cardiac calcium sparks and their molecular origins in health and disease

**DOI:** 10.1098/rsob.230045

**Published:** 2023-05-24

**Authors:** Miriam E. Hurley, Ed White, Thomas M. D. Sheard, Derek Steele, Izzy Jayasinghe

**Affiliations:** ^1^ School of Biomedical Sciences, Faculty of Biological Sciences, University of Leeds, Leeds LS2 9JT, UK; ^2^ School of Biosciences, Faculty of Science, The University of Sheffield, Sheffield S10 2TN, UK

**Keywords:** ryanodine receptor, nanodomains, calcium signalling, DNA-PAINT, correlative light microscopy

## Abstract

Rapid release of calcium from internal stores via ryanodine receptors (RyRs) is one of the fastest types of cytoplasmic second messenger signalling in excitable cells. In the heart, rapid summation of the elementary events of calcium release, 'calcium sparks', determine the contraction of the myocardium. We adapted a correlative super-resolution microscopy protocol to correlate sub-plasmalemmal spontaneous calcium sparks in rat right ventricular myocytes with the local nanoscale RyR2 positions. This revealed a steep relationship between the integral of a calcium spark and the sum of the local RyR2s. Segmentation of recurring spark sites showed evidence of repeated and triggered saltatory activation of multiple local RyR2 clusters. In myocytes taken from failing right ventricles, RyR2 clusters themselves showed a dissipated morphology and fragmented (smaller) clusters. They also featured greater heterogeneity in both the spark properties and the relationship between the integral of the calcium spark and the local ensemble of RyR2s. While fragmented (smaller) RyR2 clusters were rarely observed directly underlying the larger sparks or the recurring spark sites, local interrogation of the channel-to-channel distances confirmed a clear link between the positions of each calcium spark and the tight, non-random clustering of the local RyR2 in both healthy and failing ventricles.

## Graphical abstract

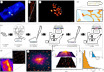

## 1. Introduction

Clusters of ryanodine receptors (RyR2) form some of the most ubiquitous intracellular calcium (Ca^2+^) signalling nanodomains in excitable cells [1–5]. The fast Ca^2+^ signals generated can trigger a range of cellular functions including gene transcription [6] and secretion [7,8], drive cellular plasticity, and modulate neuronal excitability [9] and muscle contraction [10]. This clustering is particularly critical to the Ca^2+^-induced Ca^2+^ release from the internal stores (primarily the endoplasmic reticulum; hereafter ER) into the cytoplasm via type-2 RyR. In striated muscle, elementary Ca^2+^ release events, ‘Ca^2+^ sparks’, summate to give rise to the fast and steep Ca^2+^ transients that activate cellular and, in turn, tissue contraction [11].

Ca^2+^ sparks have been observed as localized and brief elevations of cytoplasmic Ca^2+^ concentration ([Ca^2+^]*_i_*) and commonly recorded with either confocal [11,12], total internal reflection fluorescence (TIRF) [13] or types of selective plane illumination microscopies [14] for well over three and a half decades. While the size, amplitude and duration of Ca^2+^ sparks vary between cell types, [2,11] in striated muscle, they serve as a useful intrinsic bioassay of the performance of the Ca^2+^ handling and the excitation–contraction coupling which it underpins. Deviations in the physical properties of evoked or spontaneous sparks, such as width, duration, latency, shape, integrated [Ca^2+^]_i_ and frequency have often been studied as evidence of dysfunction of the cellular Ca^2+^ handling in cardiac [15–18] or skeletal [19] muscle pathologies.

Visualization of the origins of myocardial Ca^2+^ sparks, the RyR2, has advanced considerably in recent years. RyR2 clusters were first visualized as electron-dense ‘feet’ with freeze-fracture [1] and transmission electron microscopy (EM) [1,20]. Over the past two decades, the spatial organization of RyR2 clusters and the molecular components intrinsic to the nanodomains have been described through fluorescence [21,22] and tomographic EM [23–25]. The arrival of super-resolution microscopy and allied functionalities such as target counting, multiplexing and proximity detection have revealed a far more diverse and complex RyR2 cluster organization pattern in healthy and failing hearts (see review [26]). Earlier super-resolution techniques, known by acronyms such as STORM and STED, revealed not only a broader range of RyR2 cluster sizes, but also the local grouping of sub-clusters (termed ‘super clusters’) which could be functionally coupled by local Ca^2+^ gradients [17,27]. Some of the second-generation super-resolution microscopy methods that offer resolutions of 15–10 nm, chiefly DNA-point accumulation in nanoscale topography (DNA-PAINT [28]) and expansion microscopy (ExM [29]), have allowed the localization of individual RyR2 channels within the arrays that constitute these nanodomains. From these studies, we and others have shown that RyR2 are arranged in non-uniform patterns, regulated by varying densities of modulatory proteins and may be subjected to heterogeneous post-translational modifications [30–32].

A common observation from super-resolution studies of heart pathologies is the fragmented or dissipated morphology of RyR2 clusters [17,31–34]. In addition, a diminishing co-localization of RyR2 with their primary triggers [34,35], structural and modulatory partner proteins [32,33], and the increasing heterogeneity of site-specific RyR-phosphorylation [31] have also been reported, coinciding with changes in the Ca^2+^ spark properties. These concurrent shifts in both the structure and function in disease have also renewed interest in the hypothesis that the nano-scale spatial organization of RyR2 must, at least in part, encode the properties of the elementary Ca^2+^ release events. *In silico* simulations have been instrumental in demonstrating that the fragmenting RyR2 cluster morphologies may impact spark fidelity, amplitude and time course of evoked and spontaneous sparks [31,36]. They also remain powerful tools for interrogating this structure-function relationship in the context of biochemical modification or multi-molecular partnering that can shift the underpinning determinants of Ca^2+^ sparks (e.g. RyR2 open probability and luminal [Ca^2+^] of the ER or, in muscle, sarcoplasmic reticulum; hereafter SR) [31,37,38]. A significant gap that has remained however is the experimental evidence directly correlating the spark properties observed *in situ* with the true distribution of RyR2s.

In this paper, we present a direct correlation of the Ca^2+^ sparks recorded in the sub-plasmalemmal space with the true molecular-scale organization of peripheral RyR2 by leveraging a correlative structure/function imaging protocol that we described recently [39]. We have overcome the lack of widely available mammalian models with genetically encoded reporters RyR2 or [Ca^2+^]_i_ [26] to characterize the shift in the spatial encoding of ventricular Ca^2+^ sparks in right ventricular (RV) failure resulting from pulmonary arterial hypertension (PAH).

## 2. Results

### 2.1. Pipeline for correlating RyR2 underlying peripheral Ca^2+^ sparks

**Figure 1.**
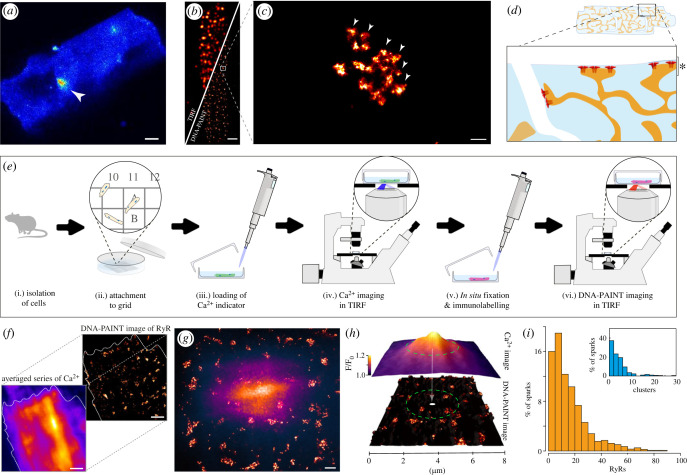
An experimental framework for spatially correlating Ca^2+^ sparks to the underlying RyR2 channel organization. (*a*) A TIRF micrograph of a spontaneous Ca^2+^ spark (white arrowhead) in the sub-surface regions of a rat right ventricular myocyte loaded with Fluo-4 AM and immersed in 5 mM [Ca^2+^]_o_. (*b*) A split view of a similar, fixed rat right ventricular myocyte immuno-stained for RyR2 imaged with standard TIRF microscopy (upper-left) and DNA-PAINT (lower-right), demonstrating the level of resolution improvement in the latter. (*c*) Magnified view of the region indicated by the box in panel (*b*) illustrating a single RyR2 cluster. The individual punctate densities of labelling (arrowheads) represented individual RyR2 channels; (*d*) A schematic illustration of a ventricular cardiomyocyte where the SR forms junctions with both the surface plasmalemma and the t-tubular invaginations. The magnified view illustrates the predominantly sub-surface RyR2 clusters observed with the thin TIRF field (depth indicated by asterisk). (*e*) Experimental pipeline developed to allow correlative Ca^2+^ spark TIRF imaging and DNA-PAINT of acutely isolated cardiomyocytes. (*f*) The image alignment involves the upscaling and registration of a time-averaged, two-dimensional version of the Ca^2+^ image series to the density-rendered super-resolution DNA-PAINT image. (*g*) An overlay between a registered Ca^2+^ spark (purple/orange colour-table) and the local super-resolution image of RyR2 clusters (red hot colour-table). (*h*) Local sampling within the ‘Ca^2+^ spark footprint’ (dashed circle) involved the interrogation of RyR2 channel and cluster position within a circular window whose diameter was equal to the average FWHM of the spark. (*i*) percentage histograms of the total RyR2 puncta counts (main panel) and of the total number of unique clusters (inset) within the ‘spark footprint’. Scale bars: (*a*) 5 µm; (*b*) 2 µm; (*c*) 50 nm; (*f*) 1 µm; (*g*) 500 nm.

Ca^2+^ sparks are commonly recorded as *x*-*t* line-scan confocal kymographs. However, the two-dimensional spatial patterns of spontaneous Ca^2+^ sparks in the sub-plasmalemmal cytoplasmic space of rat right ventricular myocytes were observable with TIRF microscopy. In TIRF image frames, sparks appear as a transient (approx. 30–100 ms) and localized event of fluorescence with a full-width-at-half-maximum (FWHM) of 1–6 µm (figure 1*a*). The current benchmark for mapping RyR2 channels in cardiomyocytes is DNA-PAINT. With approximately 10 nm of resolution, DNA-PAINT represents a significant (approx. 25-fold) improvement in optical resolution over standard diffraction-limited methods such as TIRF (figure 1*b*). With DNA-PAINT, it is now possible to resolve individual RyR2s, observed as punctate labelling densities, that make up each RyR2 cluster (figure 1*c*). In this study, we sought to selectively map the two-dimensional spatial properties of sub-plasmalemmal sparks against the local, two-dimensional organization of RyR2 located within peripheral couplons (schematically illustrated figure 1*d* and asterisked region) in healthy and failing RV myocytes using TIRF microscopy.

To this end, we developed a novel experimental pipeline detailed recently [39] (figure 1*e*). In this protocol, RV cardiomyocytes were isolated freshly from rat hearts and transferred to a glass dish with a gridded bottom, allowing the cells to adhere to the grid. Cytoplasmic fluorescent [Ca^2+^]_i_ indicator Fluo-4 AM was loaded into cells and de-esterified before cells were placed within the TIRF field. Sub-plasmalemmal Ca^2+^ sparks were recorded from cells at specific grid coordinates onto two-dimensional time series. Cells were then fixed *in situ* and labelled with anti-RyR2 antibodies towards DNA-PAINT imaging and then returned to the TIRF microscope, tracked based on their grid coordinates and DNA-PAINT series recorded. *Post-hoc* analysis included rescaling and a semi-automated alignment of a time-averaged facsimile of the Ca^2+^ time series against the DNA-PAINT image, primarily using the unique outline of the myocyte to overlay the Ca^2+^ sparks with the underlying RyR2 map (figure 1*f,g*). The coordinates of both the Ca^2+^ sparks and the individual RyR2 puncta were detected and subsequently registered against each other (using the scaling and alignment vectors established for the whole image). This allowed the RyR2 channels and clusters within the ‘spark footprint’ (determined by a circular window centred around the spark's centroid and diameter equal to the spark FWHM) to be counted and sampled on a spark-by-spark basis (figure 1*h*). The percentage histograms of the total number of RyR2 puncta (figure 1*i*; main panel) and the number of unique RyR2 clusters detected with this method in healthy RV myocytes (i.e. clusters with ≥4 RyR2 puncta; inset) within the footprint of each spark demonstrate that spontaneous Ca^2+^ sparks can arise from broadly varying ensembles of RyR2 channels (mean of apporx. 18.1 RyR2 per spark) and unique clusters (mean approx. 5.2 clusters per spark; see electronic supplementary material, figure S1A,C).

### 2.2. Correlation of RyR2 organization with Ca^2+^ sparks in RV failure

To study the structure–function relation of Ca^2+^ sparks in the failing RV, we examined myocytes acutely isolated from the RVs of rats administered with monocrotaline (MCT-RV) and age and sex-matched controls (Ctrl-RV). In the correlative, spark-by-spark analyses in MCT-RV, we observed a∼30% increase in the total number of RyR2 (mean of approx. 23.5 RyR2 per spark) and a approximately 60% increase in the number of segmented clusters (mean of∼8.1 clusters per spark; see electronic supplementary material, figure S1A–D) compared to Ctrl-RV. This was consistent with the likely recruitment of a greater number of RyR2 in the genesis of a spontaneous Ca^2+^ spark. Spontaneous sparks recorded using TIRF in MCT-RV cells also featured, on average, approximately 38% greater ‘spark mass’ (i.e. the integral of the fluorescence intensity of the spark; see electronic supplementary material, methods section for details) compared to Ctrl-RV (means approx. 38.4 compared to approx. 27.7, respectively). While the FWHM was unchanged between MCT-RV and Ctrl-RV, the spark frequency was >90% higher in MCT-RV (see plots and statistics in electronic supplementary material, figure S1E–G).

**Figure 2.**
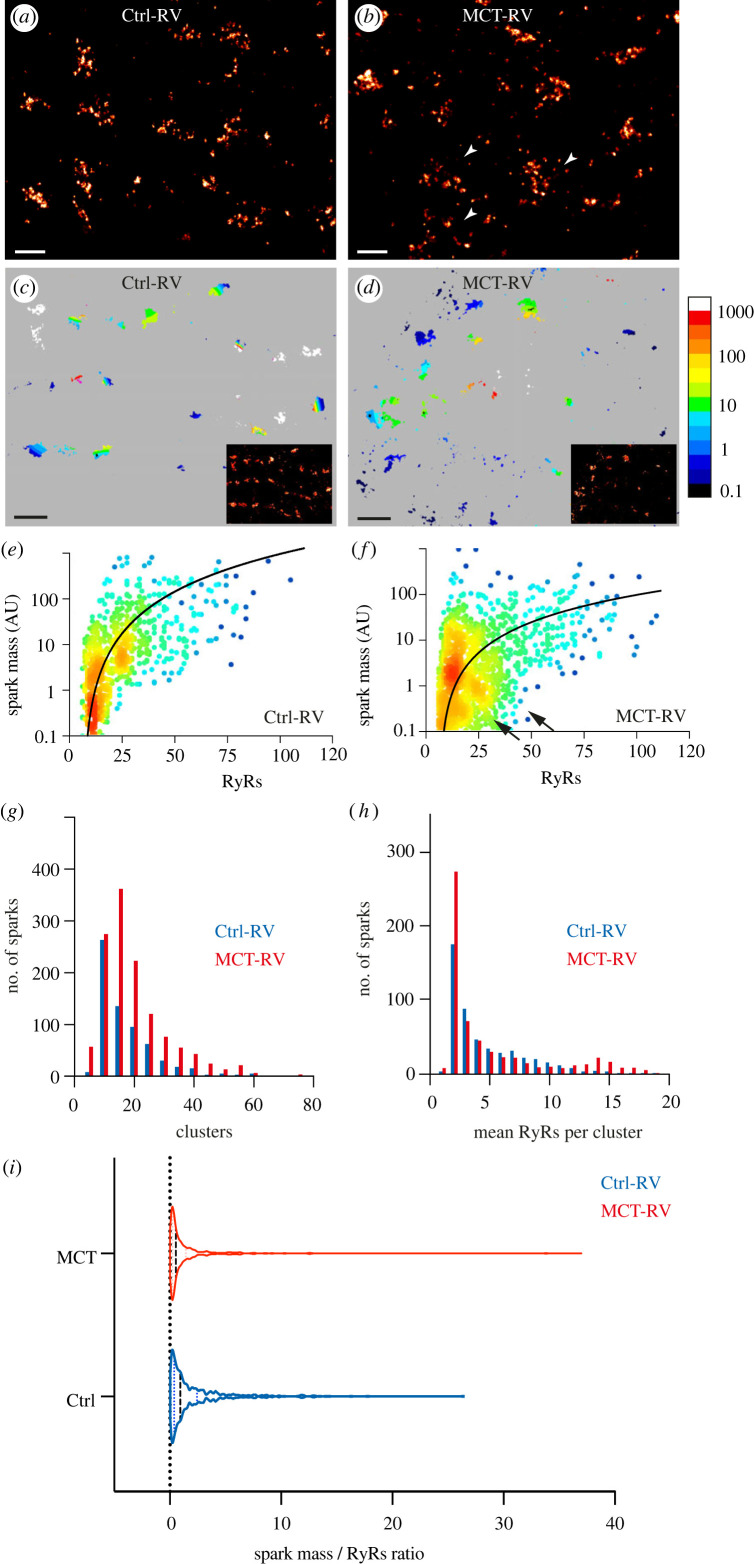
Local interrogation of RyR2 organization in Ctrl and MCT- RV cardiomyocytes. DNA-PAINT super-resolution maps of RyR2 labelling in (*a*) Ctrl and (*b*) MCT-RV rat cardiomyocytes. The arrowheads indicate the dissipated morphology of RyR2 clusters in the latter. (*c,d*) show two-dimensional super-resolution maps of RyR2, colour-coded for local, average Ca^2+^ spark mass in Ctrl-RV and MCT-RV myocytes, respectively. The insets show the original DNA-PAINT images in the corresponding region (larger versions of the insets shown in electronic supplementary material figure S2); the colour scale represents the average spark mass (in arbitrary units), estimated by xySpark. (*e,f*) Scattergrams of the Ca^2+^ spark mass, plotted on log_10_ scales against the RyR2 locally-counted within each ‘spark footprint’ in Ctrl-RV myocytes (*n* = 1050 sparks; 15 cells; 6 animals) and MCT-RV myocytes (*n* = 1529 sparks; 15 cells; 6 animals), the latter featuring sparks with smaller spark mass in regions with larger RyR2 counts. The line fits are *y* = 0.272*x*^2^ – 0.3 and *y* = 0.022*x*^2^ – 0.2 respectively. (*g*) Overlaid frequency histograms of the number of RyR2 clusters consisting of ≥5 RyR2 within the footprint of sparks recorded in Ctrl-RV (blue; *n* = 676 sparks; 6 animals) and MCT-RV (red; *n* = 1326 sparks; 6 animals). (*h*) Overlaid frequency histograms compare the distributions of mean RyR2 detected per cluster within the sparks recorded from Ctrl-RV and MCT-RV. (*i*) Shown, is a violin plot of the distribution of the ratio of spark mass to local RyR2 count recorded for each calcium spark in Ctrl-RV (blue) and MCT-RV (red).; medians (0.91 for Ctrl-RV and 0.53 for MCT-RV); mean ± s.d. 2.1 ± 3.1 for Ctrl-RV and 1.8 ± 6.7 for MCT-RV; median shown in dashed-lines and quartiles shown in dotted lines. Scale bars: (*a,b*) 200 nm; (*c,d*) 500 nm.

Close examination of DNA-PAINT images of sub-plasmalemmal RyR2 from correlative experiments showed a noticeably more dissipated morphology in MCT-RV cells compared to Ctrl-RV (figure 2*a,b*; see arrowheads in panel *b*). With the use of correlative sparks recorded over approximately 20 s, we generated ‘heat maps’ of the local nanoscale RyR2 morphology (represented by pseudocoloured binary masks of the DNA-PAINT RyR2 image; see electronic supplementary material, figure S2 for details) to indicate the average estimated spark mass of the locally recorded spontaneous Ca^2+^ sparks in Ctrl-RV and MCT-RV cells (figure 2*c,d*; insets show the greyscale DNA-PAINT images of the shown regions of interest). In the healthy control, the colour-gradations in the spark mass maps were relatively evenly distributed across clusters of similar size while smaller, fragmented clusters were sparse. In the failing cells, we observed a large majority of the fragmented (smaller and dispersed) RyR2 clusters in darker colours, suggesting that they alone are unlikely to produce sparks with greater spark mass.

The scattergrams between the spark mass of each Ca^2+^ spark recorded and the total number of RyR2 counted locally revealed a broader degree of scatter in MCT-RV compared to Ctrl-RV (figure 2*e,f*). In Ctrl-RV, sparks with spark mass <1.0 correlated with almost exclusively with regions consisting of no more than 25 RyR2 puncta. By contrast, sparks with spark mass <1.0 were correlated with a broader range of RyR2 counts (extending up to approx. 50 RyR2 puncta per spark; see the black arrows in figure 2*f*). This heterogeneity in MCT-RV is further illustrated in the frequency histograms of the total RyR2 count and the number of unique RyR2 clusters underneath each spark (figure 2*g,h*), represented by the wider distributions of MCT-RV compared to Ctrl-RV. Further, the direct ratio between the recorded spark mass and the locally counted RyR2 showed a lower mean and median for MCT-RV (mean ± s.d. 1.8 ± 6.7; median 0.53) compared to Ctrl-RV (mean ± s.d. 2.1 ± 3.1; median 0.91). The increased heterogeneity in MCT-RV was represented by an∼2.2-fold higher standard deviation compared to the control (*p* = 0.014, *B* = 6.099, d.f. = 1220 sparks; Bartlett's test; see violin plot in figure 2*i*).

### 2.3. Relationship between non-random, sub-plasmalemmal Ca^2+^ spark pattern and multi-scale RyR2 organization

**Figure 3.**
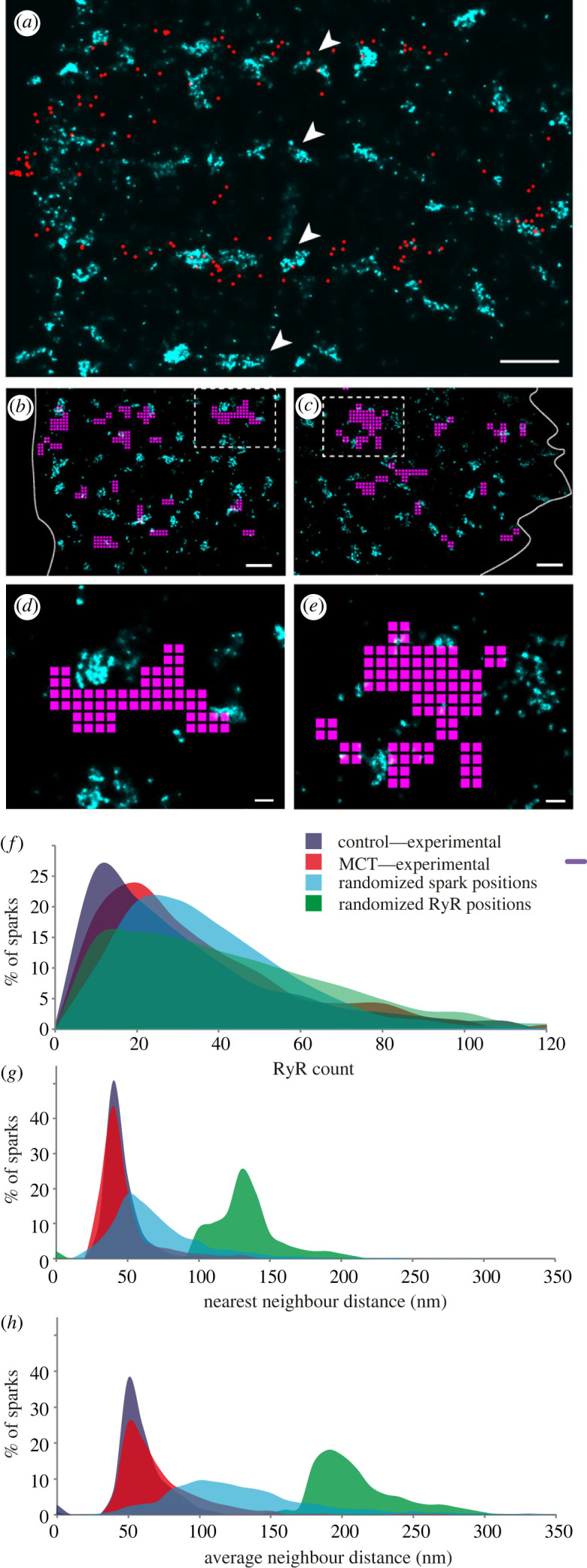
Spatial encoding of the spontaneous Ca^2+^ spark patterns in the RyR2 organization. (*a*) An overlay of an RyR2 super-resolution image (cyan) of a Ctrl-RV myocyte and a two-dimensional map of the centroids of all the spontaneous Ca^2+^ sparks (red circles) recorded within a 15 second time window. (*b,c*) Overlays of the DNA-PAINT super-resolution images of near-surface RyR2 labelling (cyan) and the recurring spark sites (magenta) in Ctrl-RV and MCT-RV myocytes respectively. (*d,e*) Magnified view of the windows indicated by dashed boxes in panels (*b,c*) respectively, illustrating that the recurring spark sites typically bridge or tessellate with local groups of RyR2 cluster rather than overlie them. (*f*) Overlaid percentage histograms of the RyR2 count underneath the spark footprint in Ctrl-RV (blue) and MCT-RV (red) as well as simulations where the detected spark positions were randomized (cyan), and where RyR2 positions were randomized. (*g*) The equivalent overlays of the percentage histograms of the mean neighbour distance between RyR2 puncta underneath each spark, and (*h*) the average distance to each of the three nearest neighbours to each RyR2 underneath spark. Scale bars: (*a–c*) 500 nm; (*d,e*) 100 nm. See electronic supplementary material, figure S4 for details of the simulation.

A novel visualization unlocked by the correlative approach is the direct overlay of the centroids of all spontaneous Ca^2+^ sparks recorded using TIRF over a window of time onto the two-dimensional super-resolution image of the intricate rows of sub-plasmalemmal RyR2 clusters (figure 3*a*). We investigated the non-random distribution of the spark centroids visually observed across the field of view by performing a two-dimensional quadtree segmentation of domains with higher density of aggregation of spark centroids over a 15 s time window (see electronic supplementary material, §S1.9 and figure S3). This segmentation revealed two-dimensional, sub-plasmalemmal regions, typically a few hundred nanometres in width, throughout a cell's footprint which we classified as ‘recurring spark sites'. Figure 3*b,c* illustrate the overlays between these recurring spark sites and the super-resolution RyR2 images in Ctrl-RV and MCT-RV respectively (magnified views shown in figure 3*d*,*e*). Strikingly, we observe that the recurring spark sites rarely overlap with larger RyR2 clusters directly. Instead, they appear to extend *between* adjacent clusters in both Ctrl-RV and MCT-RV. There was also no observable, consistent co-location of the fragmented RyR2 with the recurring spark sites in MCT-RV. The occupancy of the recurring spark sites between RyR2 clusters, often within hundreds of nanometres, resembled the shared ‘RyR2 super cluster domains’ proposed previously as a structural correlate of clusters that are likely to co-activate [40,41]. It was also conceivable that they aligned with the putative super-clusters of the Ca^2+^ release trigger, L-type Ca^2+^ channels with Ca_v_1.2 observed recently [42].

Both super-resolution and electron tomography image data have confirmed that clustering patterns of RyR2 channels in healthy cardiomyocytes, albeit not always crystalline, follow a distinct nearest-neighbour distance and the average spacings relative to the surrounding RyR2 [24,30,31]. We sought to investigate whether the spark locations are unique to regions with RyR2 clustering. To this end, we examined the above variables across experimental datasets (Ctrl-RV and MCT-RV) as well as synthetic images based on control cells where either RyR2 or Ca^2+^ sparks were positioned according to random uniform distributions (see electronic supplementary material §S1.10.1 and figure S4). A modest rightward shift and a concurrent broadening in the percentage histograms of the total RyR2 count (figure 3*f*) within each Ca^2+^ spark footprint was observed in both randomized datasets. This reinforces the gain in Ca^2+^ release that is achieved by the clustering of a relatively small number of RyR2 compared to randomly and sparsely organized RyR2 (see synthetic image in electronic supplementary material, figure S4). By contrast, considerable rightward shifts are observed in both percentage histograms of the nearest neighbour distance of each RyR2 located under each Ca^2+^ spark, and the average neighbour distance (i.e. average spacing of a given RyR2 punctum relative to the surrounding RyRs) in clusters detected underneath the spark footprint. This suggests the closeness and uniformity of the intra-cluster organization of RyR2 are intrinsic features critical to producing a spark (figure 3*g,h*). Similar to a previous report based on ExM [31], a modest shift is observed in the rightward tail in the average RyR2 neighbour distance in MCT-RV (red in figure 3*h*) which may reflect that clusters undergo loosening or ‘fraying’ of the RyR2 positions prior to the fragmentation during RV failure [32]. Generally, we note the similarity in each of these distributions between Ctrl-RV and MCT-RV datasets in comparison to the randomized datasets. This supports the RyR2 remaining within unfragmented clusters as the functionally dominant sub-population of channels driving the genesis of Ca^2+^ spark during disease.

## 3. Discussion

### 3.1. A novel approach to correlate the molecular-scale RyR2 cluster architecture with local Ca^2+^ sparks

Presented in this paper is an *experimental* interrogation of the spatial relationship between properties of the elementary events of Ca^2+^ release in primary adult cardiomyocytes and the intrinsic molecular-scale organization of RyR2. Hiess *et al.* have previously achieved a similar correlation, albeit with TIRF microscopy where GFP-RyR2 clusters were unresolved [43], hence a statistical analysis of the single-channel level RyR2 organization was not possible. Our approach was enabled by the development of a correlative imaging protocol [39] that overcomes the broad unavailability of animal models with endogenous fluorescent reporters of either RyR2 and/or [Ca^2+^]*_i_* that can simultaneously model the cellular and molecular basis of cardiomyopathy. To our knowledge, only DNA-PAINT [30] and ExM [32] currently offer optical resolution sufficient to localize individual RyR2 which motivated our choice for correlative DNA-PAINT. The ability to preferentially sample the nanoscale patterns of RyR2 organization based on a functional correlate has unlocked a powerful interrogation of the nano/micron-scale structure/function relationship.

Several key conditions and limitations in our approach are however noteworthy. The evanescent TIRF field of illumination offered us assurance that only 100–200 nm of fluorescence was being axially integrated in both the DNA-PAINT (RyR2) images and Ca^2+^ spark series. Examination of previous dSTORM [41] and 3D ExM data [32] of rat ventricular muscle that capture RyR2 clusters in both sub-plasmalemmal and interior regions offer reassurance that less than 5% of the sub-plasmalemmal clusters extend beyond this depth. While the Ca^2+^ sequences were also recorded within an evanescent field, the diffusion of both the spark Ca^2+^ and the Ca^2+^ indicator dye over micron-scale distances means that sparks originating deeper in the cell interior may in principle be visible within the TIRF field. This may limit the accuracy of the spatial correlation of the sparks and local RyR2 counts; however, our protocol to exclude sparks larger FWHM and R^2^-value (a measure of fitting accuracy and signal-to-noise ratio; SNR) from the correlative analysis allowed us to minimize the impact of out-of-plane sparks.

Deviations in the assumed flatness of these peripheral RyR2 clusters, asymmetries and heterogeneities in the two-dimensional spread of Ca^2+^ in the recorded sparks and any acute remodelling RyR2 clusters within the window of spark imaging (inside 3–4 h from the point of isolation of cells), albeit minor, are possible sources of error. While Hiess *et al.* have previously observed with confocal microscopy the movement of some peripheral RyR2 clusters in quiescent cells maintained under high [Ca^2+^]*_o_*, this fraction was reported to be small [43]. Although they could reverse this behaviour entirely with exposure to tetracaine, this strategy was incompatible with our objective of observing intrinsically spontaneous sparks. Factors that may further limit the precision of the spark-by-spark sampling of local RyR2 patterns include incomplete RyR2 labelling with the anti-RyR2 antibody chosen (used widely by many groups, and labelling efficiency previously [26] estimated to be at least approx. 90%), and the alignment error between the averaged (diffraction-limited) Ca^2+^ image and the super-resolution RyR2 image.

The key determinants of the latter are the resolution and the SNR of the averaged Ca^2+^ image. electronic supplementary material §S1.10.2 and figure S5 outline a series of simulations by which we demonstrate an alignment error under 100 nm for each iteration given the typical TIRF resolution of approximately 250 nm and SNR of approximately 17.0 in our averaged Ca^2+^ image data. For context, an alignment error of 100 nm represents the omission or addition of approximately 2.5 RyR2 channels in a tightly organized cluster in Ctrl-RV (mean nearest neighbour distance of approx. 38.23 nm). Finally, the correlative approach also prevented us from calibrating the pixel values in the Ca^2+^ images against [Ca^2+^]*_i_* with a Ca^2+^ ionophore due to the requirement of preserving the cellular structure for DNA-PAINT. As a standardization, all experiments were performed under identical excitation intensities and camera settings which allowed use to quantitatively compare the intensity information Ca^2+^ images between cells and samples.

### 3.2. Spatial encoding of sparks in the RyR2 organization

The non-random dependence of the spark locations on the close RyR2 clustering particularly reinforces numerous simulations demonstrating how close (and filled) RyR2 lattice structures promote the ignition of a spark [31,36,38]. However, similar to previous DNA-PAINT [30] and ExM [31] data, our correlative datasets feature a large majority of clusters underneath the spark footprint with looser RyR2 arrangement (typical neighbour distances >38 nm) than the fully filled lattices. As confirmed by a recent computational study, this feature is likely to contribute to the considerable variability that we see in the integrated signal of Ca^2+^ sparks [44] (e.g. Figure 2*e,f*) and certainly the broad range of ‘spark mass/RyR2s’ ratios that we observe even in the control cells.

The super-positioning of Ca^2+^ spark centroids and the RyR2 maps (e.g. figure 3*a*) provided us with an unprecedented view into the spatial encoding of the spark locations across the sarcomerically organized Ca^2+^ handling machinery. The localizations of the recurring spark sites *between* larger RyR2 clusters are, to our knowledge, the only *experimental* imaging data supporting the putative ‘triggered saltatory’ recruitment of neighbouring RyR2 clusters at the genesis of a Ca^2+^ spark [40] in both healthy and failing myocytes. While these recurring spark sites (figure 3*b–e*) may be accentuated by the possible constructive interference of concurrent sparks or waves, constraining this visualization to sparks with FWHM ≤ 6.0 µm, we have minimized the impact of such spurious localizations.

The scattergrams between spark mass and local RyR2 counts (figure 2*e,f*) demonstrate a broad correlation in both failing myocytes and the healthy controls. When the two-dimensional positions of RyR2 and sparks were randomized separately, the distribution of the total RyR2 count within the spark footprint was not entirely annulled (figure 3*f*). In fact, it became broader, suggesting that the local RyR2 ensemble is only a rough determinant of the Ca^2+^ spark. The high degree of scatter in both spark mass scattergrams of Ctrl-RV and MCT-RV (but exacerbated in sparks with higher spark mass in MCT-TV) further reflects how numerically similar pools of RyR2 can give rise to Ca^2+^ sparks whose integrated [Ca^2+^]*_i_* can vary by 2–3 orders of magnitude.

### 3.3. Heterogeneities in the structure/function relation in RV failure

Increasing structural heterogeneity, including local t-tubule remodelling [45–47], local regions of RyR2 fragmentation [17,38,48] and diminishing regularity of the expression patterns Ca^2+^ handling proteins over nanometre/micron length-scales [49], are now understood to be hallmarks of wide-ranging cardiac pathologies. In MCT-RV, we have previously observed evidence of heterogeneous RyR2 cluster fragmentation [31] and the receding sub-domains of RyR2-modulators and molecular tethers, junctophilin-2 (JPH2) [32] and BIN-1 from some clusters [18]. In this context, the increasing heterogeneity in the ratio between spark mass and RyR2 count in MCT-RV (figure 2*i*) is the likely result of differently configured *local* Ca^2+^ handling machineries, not limited to the spatial organization of RyR2. Downregulation of the cytoplasmic Ca^2+^ removal mechanisms (the Na^+^/Ca^2+^ exchanger and SR Ca^2+^ ATPase) [18,50] along with the overload of SR luminal [Ca^2+^] need to be considered with the 30% increase in the RyR2 ensemble observed underneath Ca^2+^ sparks. While the individual cluster size of RyR2 is reduced in MCT-RV as a consequence of the cluster fragmentation or fraying [31,32], the modest increases in the RyR2-RyR2 neighbour distance measurements offer some initial clues of the evolving functional coupling in and around the dyads. An enhancement of the functional coupling of the loosely packed RyR2 is certainly conceivable with the reduced L-type Ca^2+^ channel expression [50] and higher SR [Ca^2+^]*_i_* in MCT-RV myocytes, offering greater driving force during Ca^2+^ release. The super-resolution heatmaps encoding spark mass do not however reveal a clear correlation between fragmented RyR2 and regions with higher average spark mass. This observation may however be different if Ca^2+^ leak could be mapped with a more sensitive approach than diffraction-limited TIRF imaging with Fluo-4 Ca^2+^ indicator.

## 4. Material and methods

### 4.1. Microscope setup

All experiments were performed on a Nikon TE2000 (Nikon; Japan) modified to enable TIRF dual-colour imaging with 488 nm and 671 nm excitations on an approximately 15 µm × 15 µm illumination field (as detailed previously [39]). The full list of specifications and settings can be found in the electronic supplementary material, methods section. Emitted light was recorded onto a Zyla 5.5 scientific CMOS camera (sCMOS; Andor, Belfast). Raw image series for both Ca^2+^ imaging and DNA-PAINT were acquired using the open-sources Python Microscopy Environment [51] (PyME) software.

### 4.2. Animal models and isolation of ventricular myocytes

Experiments were performed according to the UK Animals (Scientific Procedures) Act of 1986 and with UK Home Office approval (license number 70/8399) and local ethical approval. As a model of right ventricular (RV) failure, adult male Wistar Crl rats aged approximately 5 weeks were given an intraperitoneal injection of crotaline (Merck, NJ) to induce pulmonary arterial hypertension. Age and sex-matched controls were given an equivalent bolus of saline. At 3–4 weeks, the MCT treated animals were monitored for signs of RV failure were euthanized along with age-matched Ctrl animals. Hearts were dissected acutely and right ventricular myocytes enzymatically isolated following Langendorff perfusion. For a detailed account of the animal model and cell isolation, see the electronic supplementary material, methods section.

### 4.3. Ca^2+^ sparks imaging

RV myocytes were loaded with Fluo-4 AM fluorescent Ca^2+^ indicator (ThermoFisher Scientific) and immersed in a Tyrode's solution containing 5 mM CaCl_2_ at pH 7.4 and adhered to a gridded imaging dish with a #1.5H glass coverslip bottom (Ibidi, USA) for 90 mins before being immersed in fresh Tyrode's solution for Ca^2+^ spark imaging. The dishes were clamped securely onto the stage of the TIRF microscope system such that the grid was aligned with the straight edges of the camera's field of view, under brightfield illumination. Cells forming a substantial footprint were illuminated with a 488 nm laser. The local changes in the Fluo-4 fluorescence were recorded at a frame rate of 10 Hz with either Tetraspeck microspheres (ThermoFisher Scientific) attached to the imaging grid or the outline of the cell's contact patch with the grid included within the imaging frame. See detailed protocol in the electronic supplementary material, methods section.

### 4.4. Cell fixation and immunolabelling for DNA-PAINT imaging of RyR2

Immediately following Ca^2+^ imaging, cells were fixed *in situ* with 2% paraformaldehyde (Sigma-Aldrich; w/v in phosphate buffered saline; PBS) for 10 mins at room temperature (RT). Samples were washed and subjected to immunofluorescence labelling with a monoclonal mouse anti-RyR2 IgG (MA3–916; ThermoFisher) primary antibody and an anti-mouse IgG secondary antibody (Jackson Immunoresearch) conjugated to a DNA-PAINT P1 ‘docking’ strand (as designed by Jungmann *et al.* [28]) was applied, diluted at 1 : 100 in incubation solution. See details of the labelling protocol and the production of the secondary antibody and the base sequences of the P1 ‘docking’ strands in the electronic supplementary material, methods section.

### 4.5. DNA-PAINT imaging and primary processing

Following immunolabelling, samples were washed 3 times in ‘Buffer C’ formulated by Jungmann *et al.* [28], and the P1 ‘imager’ strands linked to Atto655 were applied at 1.2 nM in Buffer C. The dish was returned to the microscope stage and the grid coordinates recorded from the Ca^2+^ imaging were used to return the field of view to the corresponding cells imaged previously. For exciting the Atto655 on the imager strands marking the RyR2 targets at the very edge of the cell, the 671 nm laser was focused at a supra-critical angle onto the field of view to achieve evanescent TIRF illumination. Time series of the DNA-PAINT events consisting of 20 000–50 000 frames were acquired at 100 ms/frame integration time. The analysis included the detection of single molecule events and least-squares fitting of a two-dimensional Gaussian to localize their sub-pixel scale centroid. The event positions were then rendered into a 16-bit greyscale TIFF image with a pixel scaling of 5 nm per pixel using an algorithm based on Delaunay triangularization [52].

### 4.6. Correlative and quantitative image analysis

The full image analysis protocol consisting of the registration of the Ca^2+^ spark data to the DNA-PAINT data and the localization of Ca^2+^ sparks has been published elsewhere [39].

#### 4.6.1. Ca^2+^ spark detection

The Ca^2+^ spark localization tool ImageJ plugin software: ‘xySpark’ [12] was used for background estimation, detection and localization of individual Ca^2+^ sparks. Output of this analysis was a list of *x*, *y* and *t* coordinates of each spark along with their full-width at half maximum (FWHM) estimated by the Gaussian fit, coefficient of determination *R*^2^, amplitude estimated as *F*/*F*_0_, where *F*_0_ was the estimate of the baseline level of the Ca^2+^ indicator fluorescence in a local cytoplasmic region and *F* was the fluorescence intensity value at the peak of the spark. Only sparks with 1.0 µm ≤ FWHM ≤ 6.0 µm (to select for in-focus sparks) and an *R*^2^ value ≤ 0.5 (sparks with a sufficiently high signal-to-noise ratio for non-spurious localization) were filtered and retained for further analysis, as established previously by Hurley *et al.* [39].

#### 4.6.2. RyR2 puncta localization

The punctate RyR2 labelling densities in the rendered images were detected using a centroid detection algorithm available through PyME and describe previously [30]. The list of coordinates from this analysis were used for the correlative analysis of RyR2, analysis of neighbour distances between local RyRs, and counting RyR2 numbers within cluster and underneath the spark footprints.

#### 4.6.3. Image correlation and alignment of discretized Ca^2+^ sparks and RyR2 puncta

We used an image alignment pipeline code to upscale the Ca^2+^ images and align the coordinates of the Ca^2+^ sparks to the DNA-PAINT RyR2 image, as described recently [39]. Briefly, approximately 10 consecutive frames from the Ca^2+^ time series were averaged to produce a low-noise, diffraction-limited image of the cell. This image was taken as a reference image of the cell's relative position against the Tetraspeck microsphere fiduciary markers and the cell boundaries. The code upscaled the low-noise Ca^2+^ image to match the pixel scaling of the DNA-PAINT image. It then required the user to manually align the Ca^2+^ image against the DNA-PAINT image, using either the cell outlines and/or the Tetraspeck fiduciary markers as guides. The *x* and *y* shift coordinates used for this alignment were then used as a starting point to perform an automated fine-alignment through cross-correlation of the images. The shift coordinates determined through the fine alignments were then applied to the Ca^2+^ spark coordinates align them against the maps of RyR2 puncta. See Hurley *et al.* [39] for details.

#### 4.6.4. Local sampling of RyR2 organization using Ca^2+^ spark footprint

RyR2 centroids and/or segmented RyR2 clusters located inside a circular window whose diameter was equal to the FWHM of the spark was included in the correlative analysis for each spark. In correlation of the RyR2 count beneath each spark against ‘spark mass’, the latter was calculated as the product of the spark amplitude (*F*/*F*_0_), FWHM^3^ and conversion factor 1.206, and was one of the default output parameters of the xySpark software [53]. Data points in scattergrams (figure 2*e,f*) represent individual sparks (taken as technical replicates). Biological replicates (cells and animals) are also stated in Figure legends (figure 2 and electronic supplementary material, figure S1). In the analysis of NND and 3ND, only RyR2 clusters (as defined by each segmented area) that consisted four or more RyR2 were considered. The NND value for each spark sampled represented the average of the distance from each RyR2 centroid to its nearest neighbour. 3ND was the average of the distances from each RyR2 centroid to each of its three nearest neighbours. The mean of all 3ND estimates for all RyR2 found underneath each spark is shown in figure 3 as a measure of the overall uniformity of RyR2 arrangement within their clusters, as introduced previously [26,31].

#### 4.6.5. Cluster segmentation

Using custom-written programs implemented in IDL, a global threshold which encapsulated 80% of the total labelling fraction above background was adopted to generate a mask of the RyR2 labelled area defining each cluster as per previously published studies [26,31].

#### 4.6.6. Statistical tests

Data comparing RyR2 counts and spark properties did not meet the criteria for parametric tests; therefore the comparisons between MCT-RV and Ctrl-RV represent Mann–Whitney *U* tests (see electronic supplementary material, figure S1).

## Data Availability

The primary code used for the correlative analysis is available via GitHub repository https://github.com/ijayas/imagealigning/ and original description of the methodology by Hurley *et al*. [39]. Implementation of the code is available through the scripts included in the data supplement. The data are provided in the electronic supplementary material [54].
